# A combined nomogram based on radiomics and hematology to predict the pathological complete response of neoadjuvant immunochemotherapy in esophageal squamous cell carcinoma

**DOI:** 10.1186/s12885-024-12239-0

**Published:** 2024-04-12

**Authors:** Yu Yang, Yan Yi, Zhongtang Wang, Shanshan Li, Bin Zhang, Zheng Sang, Lili Zhang, Qiang Cao, Baosheng Li

**Affiliations:** 1 Shandong Medical Imaging and Radiotherapy Engineering Center (SMIREC), Shandong Cancer Hospital and Institute, Shandong First Medical University and Shandong Academy of Medical Sciences, Jinan, China; 2 Department of Radiation Oncology, Shandong Cancer Hospital and Institute, Shandong First Medical University and Shandong Academy of Medical Sciences, Jinan, China; 3 Department of Oncology, Yantai Affiliated Hospital of Binzhou Medical University, Yantai, China

**Keywords:** Pathological complete response, Neoadjuvant immunochemotherapy, Nomogram, Radiomics, Hematology, Esophageal squamous cell carcinoma

## Abstract

**Background:**

To predict pathological complete response (pCR) in patients receiving neoadjuvant immunochemotherapy (nICT) for esophageal squamous cell carcinoma (ESCC), we explored the factors that influence pCR after nICT and established a combined nomogram model.

**Methods:**

We retrospectively included 164 ESCC patients treated with nICT. The radiomics signature and hematology model were constructed utilizing least absolute shrinkage and selection operator (LASSO) regression, and the radiomics score (radScore) and hematology score (hemScore) were determined for each patient. Using the radScore, hemScore, and independent influencing factors obtained through univariate and multivariate analyses, a combined nomogram was established. The consistency and prediction ability of the nomogram were assessed utilizing calibration curve and the area under the receiver operating factor curve (AUC), and the clinical benefits were assessed utilizing decision curve analysis (DCA).

**Results:**

We constructed three predictive models.The AUC values of the radiomics signature and hematology model reached 0.874 (95% CI: 0.819–0.928) and 0.772 (95% CI: 0.699–0.845), respectively. Tumor length, cN stage, the radScore, and the hemScore were found to be independent factors influencing pCR according to univariate and multivariate analyses (*P* < 0.05). A combined nomogram was constructed from these factors, and AUC reached 0.934 (95% CI: 0.896–0.972). DCA demonstrated that the clinical benefits brought by the nomogram for patients across an extensive range were greater than those of other individual models.

**Conclusions:**

By combining CT radiomics, hematological factors, and clinicopathological characteristics before treatment, we developed a nomogram model that effectively predicted whether ESCC patients would achieve pCR after nICT, thus identifying patients who are sensitive to nICT and assisting in clinical treatment decision-making.

**Supplementary Information:**

The online version contains supplementary material available at 10.1186/s12885-024-12239-0.

## Background

The incidence rate of esophageal cancer is the seventh-highest worldwide among all cancers, and esophageal squamous cell carcinoma (ESCC) was the diagnosis made in more than 90% of cases in China [1, 2]. The primary therapeutic method for locally advanced ESCC is surgery, but it has not yet achieved satisfactory outcomes. To increase patient survival rates, neoadjuvant treatments such as targeted therapy, chemotherapy, radiotherapy, and immunotherapy were introduced. Several clinical studies on neoadjuvant immunochemotherapy (nICT) for locally advanced ESCC are ongoing. Preliminary findings indicated that esophagectomy for esophageal cancer after nICT showed favorable efficacy and safety, and patients who received nICT exhibited satisfactory pathological complete response (pCR) and R0 resection rates [3–6]. The prognostic survival for patients with pCR is much better than that of non-pCR and may be able to delay or avoid surgery, thus demonstrating the great promising nICT is as a neoadjuvant treatment for ESCC [7–9].

Only 20%-40% of ESCC patients achieve a pCR after nICT because of tumor heterogeneity [6, 10, 11]. In contrast, many patients not only end up without obvious treatment reactions and cannot achieve practical benefits but will also experience the costly and dangerous side effects of medications. Therefore, the prediction of treatment response significantly affects the implementation of nICT. Relevant research reports show that tumor mutational burden, the ratio of monocytes to lymphocytes, PD-1/PD-L1 expression, high microsatellite instability, and other biomarkers are associated with immunotherapy effectiveness [12–14]. There are also reports that inflammatory markers, circulating lymphocyte subtypes, and numbers of blood cells are strongly linked to patient survival and therapeutic response [15]. Unfortunately, there are no reliable biomarkers to forecast how ESCC patients would respond to therapy following nICT.

Radiomics is a high-throughput method for obtaining a great deal of data from images. Through deeper mining of massive data, radiomics can provide a series of valuable auxiliary treatment methods for the diagnosis of disease [16], the evaluation of therapeutic response [17], and the prediction of lymph node metastasis [18]. Previous studies have reported that radiomics is an efficient way for predicting the therapeutic response of ESCC after neoadjuvant chemoradiotherapy (nCRT) [19] and can aid in making clinical treatment decisions. Biomarkers obtained from biopsy require invasive examination and a series of time-consuming and complex laboratory procedures. However, it is more valuable to explore noninvasive biomarkers for predicting the therapeutic response of ESCC patients after nICT.

A nomogram is a numerical estimation model that includes many complex factors that can clearly and intuitively predict the probability of event occurrence. Numerous nomogram models for prediction have been constructed and are frequently utilized in cancer prediction modeling [20, 21]. To predict the pCR of ESCC patients after nICT, we explored the factors that influence pCR after nICT and established a combined nomogram model.

## Methods

### Study design

**Fig. 1:**
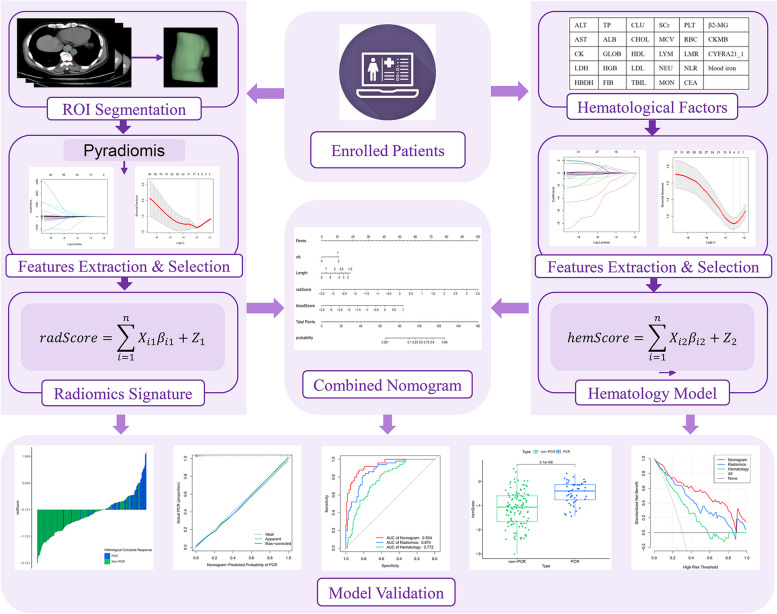
The research workflow. After the patient was enrolled, radiomics and hematology analyses were conducted separately, using LASSO regression to reduce the dimensionality of features. Then, radScore and hemScore were calculated, and they were combined with independent factors influencing pCR to develop a nomogram. Evaluated the predictive ability of three models, including ROC curve, calibration curve, DCA, etc.

Fig. 1 shows the research procedure, which is comprehensively described by the following methods.

### Patients

**Fig. 2:**
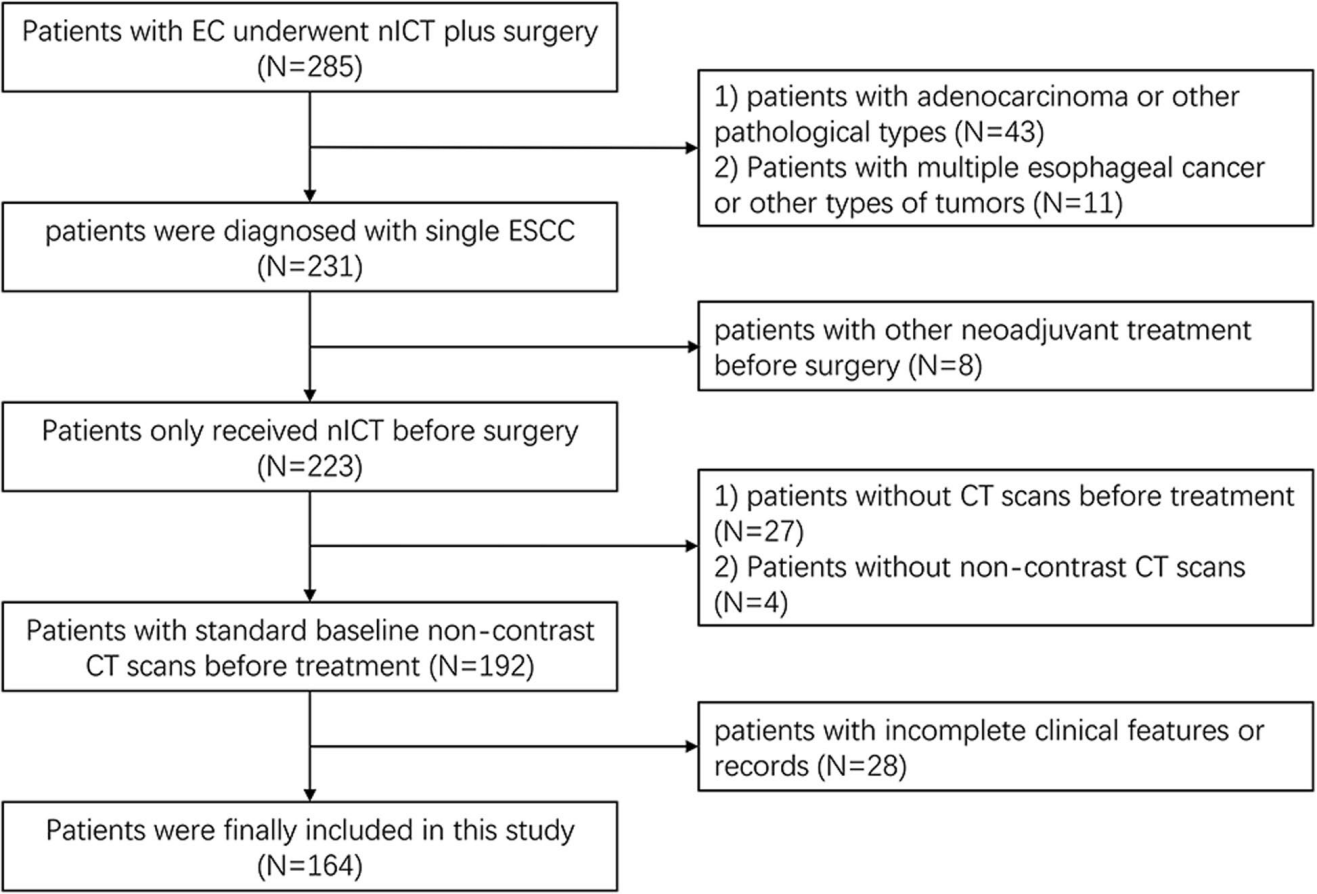
Flowchart of patient enrollment

This study retrospectively collected 285 patients who received nICT along with esophagectomy at our hospital from September 2020 to April 2023. The institutional review board (IRB) of Shandong Cancer Hospital and Institute authorized our study (approval number: SDTHEC2023001010), and the IRB exempted informed consent. The inclusion criteria for patients were as follows: (a) verified by histopathology as ESCC; (b) standard non-contrast chest CT scan before treatment; and (c) received nICT and underwent esophagectomy. The exclusion criteria for patients were as follows: (a) treated with chemotherapy, radiotherapy, or other anticancer therapies prior to the baseline CT scans; (b) had other concurrent tumors; (c) had incomplete clinical features or records; and (d) had poor-quality CT images or primary tumors that were too small to be identified. There were 164 patients included after applying the inclusion and exclusion criteria (Fig. 2).

### Treatment protocol

Every patient received 2–4 cycles of immunochemotherapy and underwent open or laparoscopic thoracoscopic McKeown surgery at 4–6 weeks after treatment. The chemotherapy regimens used were fluorouracil (5-FU, teysuno, or capecitabine) or paclitaxel (albumin-binding paclitaxel or docetaxel) combined with platinum (cisplatin, nedaplatin, or oxaliplatin). A PD-1 inhibitor was chosen for the immunotherapy regimen (camrelizumab, tislelizumab, pembrolizumab, toripalimab, or sintilimab). The combination regimen and dosage depended on the patient's actual situation and preferences.

### Data collection

Before patients received nICT, 40 potential influencing factors were retrospectively collected from electronic medical records. These indicators were divided into clinicopathological factors (*n* = 11) and hematological factors (*n* = 29). The clinicopathological factors included sex, age, smoking history, drinking history, tumor length, tumor location, tumor differentiation, cT stage, cN stage, cTNM stage (AJCC 8th edition), and Karnofsky performance status (KPS) score. The hematological factors are displayed in Table S1. The outcome was whether the patient achieved pCR. Based on the AJCC (8th edition) tumor regression grade (TRG), pCR was defined as follows: (1) original tumor without viable tumor cells (TRG 0); and (2) no positive lymph nodes in the surgical specimens.

### CT image acquisition and ROI segmentation

In our study, due to the well-differentiated tumor borders, non-contrast CT images before nICT were utilized for tumor segmentation and feature extraction. Each patient received a standard Philips CT scanner chest scan (Brilliance iCT 128). The following scanning protocol was used: 5-mm-thick slice reconstructions, helical scanning mode, 120 kV tube voltage, and 300–500 mA tube current.

With the patient information hidden, 2 radiologists with over 5 years of expertise performed the original tumor segmentation. Because human observations are susceptible to differences from person to person, a single radiologist segmented 100 randomly selected ESCC patients one month later to assess and confirm the repeatability of radiomics features. 2 radiologists reached a consensus on the differences in regions of interest (ROIs) through negotiation, and patients in which the radiologists were unable to reach an agreement on were excluded. All ROIs were segmented utilizing a 3D slicer (version 5.2.1).

Significantly, the clinical stage (cT stage, cN stage, and cTNM stage) of patients was determined separately by oncologists based on their gastroscopy and contrast CT, while non-contrast CT was used only for radiomics feature extraction.

### Radiomics feature extraction and selection

We first performed preprocessing by resampling all ROIs to 1 × 1 × 1 mm^3^ and areas with HU < 0 were excluded. Radiomics features were subsequently extracted from the preprocessed ROIs utilizing PyRadiomics (version 3.6.2). The radiomics features we extracted included the following categories: first-order features, shape features, second or higher-order texture features, and the features were based on the original image obtained through the Laplace transform and wavelet transform.

Feature selection consisted of two steps: first, after preprocessing all the radiomics feature values via Z score normalization, the repeatability of radiomics features was assessed through intraclass correlation coefficient (ICC) analysis. Only the features with a *P*-value < 0.05 and an ICC ≥ 0.9 were screened for the next step of the analysis. Then, R software (version 4.0.6) was used to perform least absolute shrinkage and selection operator (LASSO) regression to identify features related to pCR, and the optimal parameter (λ) was chosen by five-fold cross-validation. In the process of selecting features, LASSO regression incorporates L1 regularization, which utilizes gradient optimization to fine-tune parameter coefficients, thereby balancing their relative weights and mitigating the issues of overfitting and collinearity. Due to the imbalance of our dataset, we also utilized the synthetic minority oversampling technique (SMOTE) in our analysis to balance the number of patients with non-pCR against pCR [22].

### Construction and evaluation of the radiomics signature, hematology model, and combined nomogram

First, a radiomics signature and a hematology model were developed using the LASSO method. Calculated the radiomics score (radScore) and hematology score (hemScore) for each patient by linearly combining the coefficient weighting of the selected features. Second, the univariate and multivariate analyses were utilized to screen out prognostic variables with P values < 0.05 for clinicopathological factors. Finally, by merging the radScore, hemScore, and clinicopathological independent factors, a combined nomogram model was constructed. The receiver operating characteristic (ROC) curve was utilized to estimate the precision of the combined nomogram, and the consistency of the combined nomogram was verified using a calibration curve. Decision curve analysis (DCA) was utilized to fully estimate and distinguish the clinical net benefit rates of the three models.

### Statistical analysis

To assess the differences in clinical characteristics among non-pCR and pCR patients, continuous variables were examined utilizing the Student's t-test, while categorical variables were examined utilizing the chi-square test or Fisher's exact test. The model's ability for predicting was estimated utilizing the area under the ROC curve (AUC) and DeLong test. The calibration curve was utilized to fully estimate the consistency of the model, and DCA was utilized to compare and estimate the clinical net benefit rates of the three models. Whether the predicted results of the combined nomogram fit the actual situation was determined using the Hosmer–Lemeshow test. All the statistical analyses and graphical plots were generated utilizing R software (version 4.0.6). A P-value < 0.05 is defined as having statistical differences.

## Results

### Patient characteristics

The baseline clinicopathological characteristics of the 164 eligible ESCC patients were displayed in Table 1. The results revealed significant differences among non-pCR patients and pCR patients in tumor length (*P* = 0.025), cN stage(*P* = 0.040), and age (*P* = 0.041).

**Table 1:** Clinicopathological characteristics of the patients

Characteristic	pCR	non-pCR	p-value
	**(n = 50)**	**(n = 114)**	
Sex, n. (%)			0.151
Female	10 (20.0)	13 (11.4)	
Male	40 (80.0)	101 (88.6)	
Age, n. (%)			0.041*
≤ 60	15 (30.0)	54 (47.4)	
> 60	35 (70.0)	60 (52.6)	
cT stage, n. (%)			0.691
2	5 (10.0)	7 (6.1)	
3	41 (82.0)	97 (85.1)	
4	4 (8.0)	10 (8.8)	
cN stage, n. (%)			0.040*
0	4 (8.0)	28 (24.6)	
1	33 (66.0)	60 (52.6)	
2	13 (26.0)	26 (22.8)	
cTNM stage, n. (%)			0.224
II	8 (16.0)	32 (28.1)	
III	38 (76.0)	72 (63.2)	
IV	4 (8.0)	10 (8.8)	
KPS score, n. (%)			0.463
80	33 (66.0)	82 (71.9)	
90	17 (34.0)	32 (28.1)	
Smoking history, n. (%)			1.000
No	23 (46.0)	51 (44.7)	
Yes	27 (54.0)	63 (55.3)	
Drinking history, n. (%)			0.614
No	21 (42.0)	53 (46.5)	
Yes	29 (58.0)	61 (53.5)	
Tumor location, n. (%)			0.852
Lower	31 (62.0)	70 (61.4)	
Middle	17 (34.0)	36 (31.6)	
Upper	2 (4.0)	8 (7.0)	
Tumor differentiation, n. (%)			0.576
High	6 (12.0)	8 (7.0)	
Low	18 (36.0)	42 (36.8)	
Middle	26 (52.0)	64 (56.1)	
Tumor length, mean (SD)	4.5 (1.3)	5.1 (1.4)	0.025*

Note. *pCR* pathological complete response, *cT* clinical T category, *cN* clinical N category, *cTNM* clinical TNM category, *KPS* Karnofsky performance status, * represented statistical significance

### Construction and validation of radiomics signature

From every ROI, we extracted 1046 radiomics features. 8 optimal radiomics features (Table 2) related to pCR were identified through LASSO regression, and a radiomics signature was constructed to predict whether patients would achieve pCR after treatment. LASSO dimensionality reduction and the corresponding cross-validation procedures in radiomics are shown in Fig. S1. The radScore was calculated with the following formula:

**Table 2:** LASSO regression selected radiomics feature linked to pCR

Radiomics features	Coefcients
log.sigma.4.0.mm.3D_firstorder_Median	0.002644
log.sigma.4.0.mm.3D_glrlm_LowGrayLevelRunEmphasis	1.064868
log.sigma.4.0.mm.3D_glszm_GrayLevelVariance	-0.00517
wavelet.LHH_firstorder_Skewness	-0.42175
wavelet.LHH_glcm_Imc2	-15.2844
wavelet.HLL_glcm_InverseVariance	3.030031
wavelet.HLH_glrlm_RunVariance	1.097568
wavelet.LLL_glcm_ClusterShade	-0.00371

$$radScore=\sum_{i=1}^{n}{X}_{i1}{\beta }_{i1}+{Z}_{1}$$

${X}_{i1}$ is the radiomics feature determined through LASSO regression, ${\beta }_{i1}$ is the regression coefficient of ${X}_{i1}$, and ${Z}_{1}$ is a constant term.

**Fig. 3:**
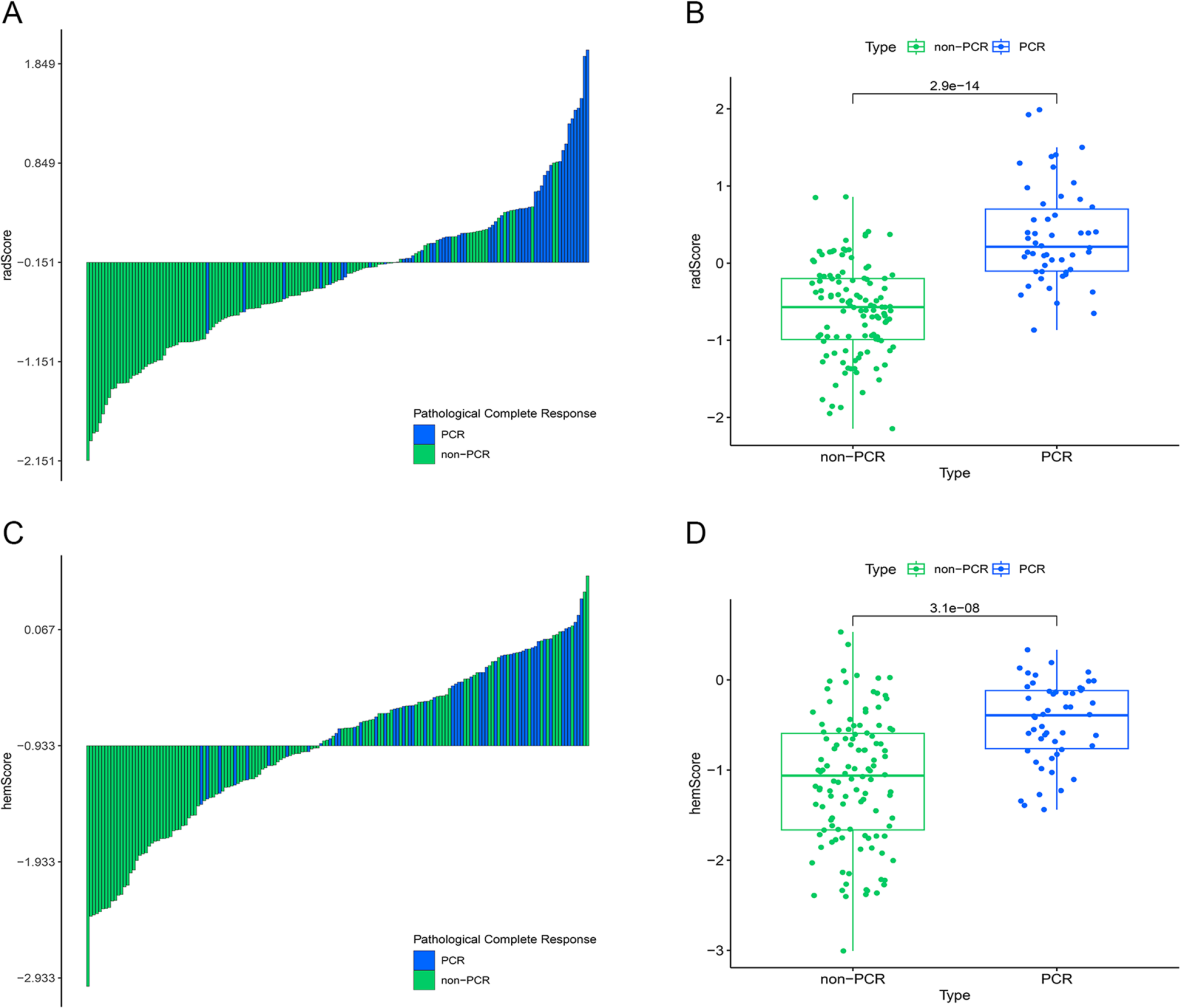
Radiomics signature and hematology model predictive performance. A: Radiomics signature value bar plot for every ESCC patient. The optimal threshold value for distinguishing among cohorts with non-pCR versus pCR is -0.151. B: Comparison of radScore among non-pCR versus pCR cohorts (*P* < 0.001). C: Hematology model value bar plot for every ESCC patient. The optimal threshold value for distinguishing among cohorts with non-pCR versus pCR is -0.933. D: Comparison of hemScore among non-pCR versus pCR cohorts (*P* < 0.001). The green color in the figure represents non-pCR patients, while the blue color represents pCR patients

A threshold of -0.151 was determined by the Youden index to differentiate among cohorts with non-pCR versus PCR (Fig. 3A). In terms of the actual classification, there was a difference in statistics in the radScore among cohorts with non-pCR versus pCR (*P* < 0.001) (Fig. 3B). The pCR of patients can be reliably predicted by the radiomics signature, and the AUC reached 0.884 (95% CI: 0.841–0.927) for balanced data with SMOTE and 0.874 (95% CI: 0.819–0.928) for actual data (Fig. S2). The P value of the DeLong test between the two cohorts was 0.772, indicating no significant difference. Then, we used actual data to calculate the radScore and included it in subsequent analyses.

### Construction and validation of hematology model

We identified 29 hematology indicators as potential influencing factors for predicting the acquisition of a pCR after nICT. LASSO regression revealed that lymphocyte count (LYM), high-density lipoprotein (HDL), albumin (ALB), and neutrophil-to-lymphocyte ratio (NLR) had a bearing on pCR, and a hematology model was constructed (Fig. S3, Table S2). The hemScore was calculated with the following formula:

$$hemScore=\sum_{i=1}^{n}{X}_{i2}{\beta }_{i2}+{Z}_{2}$$

${X}_{i2}$ is the hematology factor identified by LASSO regression, ${\beta }_{i2}$ is the regression coefficient of ${X}_{i2}$, and ${Z}_{2}$ is a constant term.

**Fig. 4:**
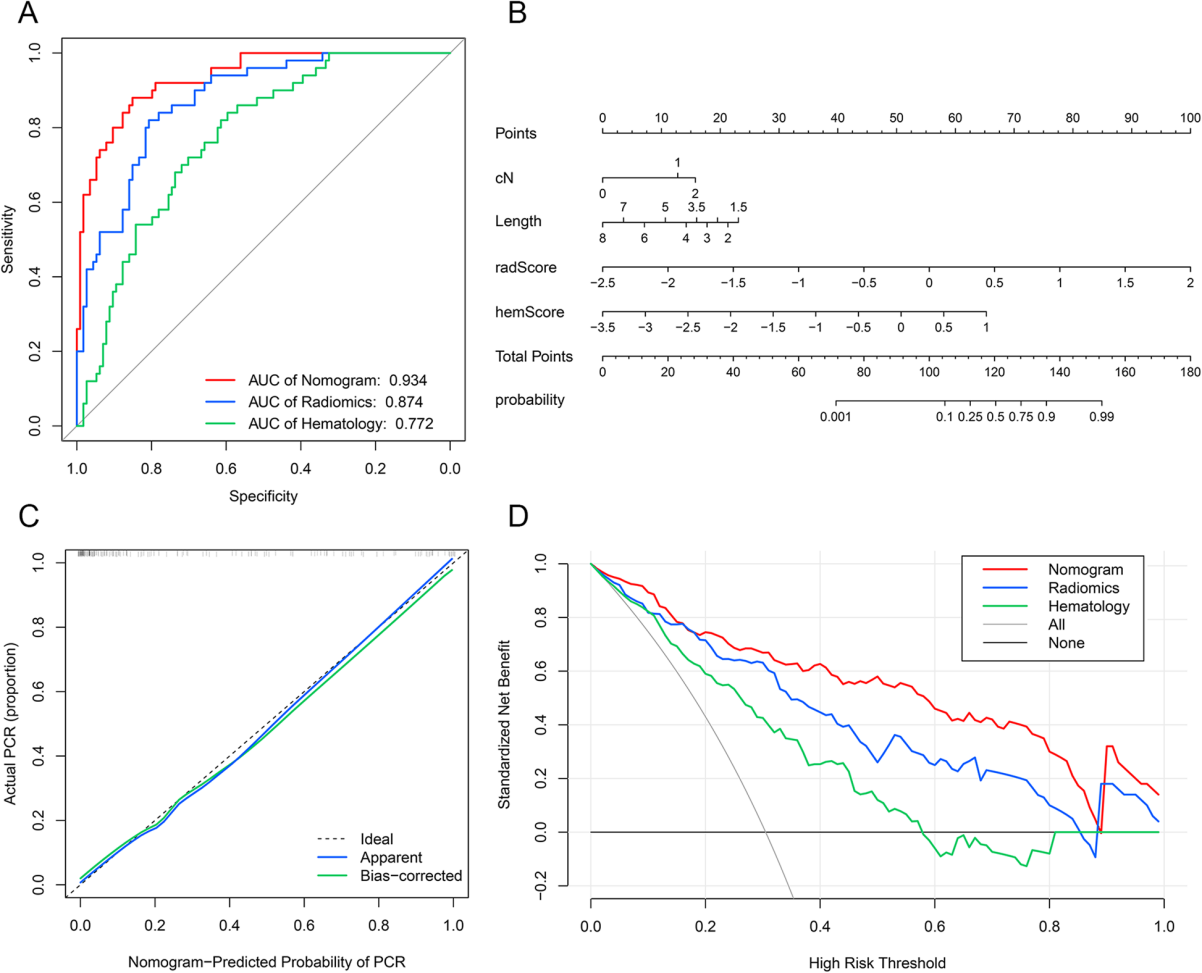
Comparison of predictive performance of the combined nomogram, radiomics signature, and hematology model. A: Receiver operating characteristic curves of the combined nomogram, radiomics signature, and hematology model. B: Nomogram based on independent predictors (radScore, hemScore, cN stage, and tumor length). C: Calibration curves of the combined nomogram to estimate the consistency among the estimated pCR probability by the combined nomogram and the authentic pCR. The ideal situation is shown by the black dashed line, which acts as the reference line and shows when the predicted and actual values coincide; the actual situation of the nomogram is shown by the solid blue line, referred to as the apparent line; the bias-corrected line is shown by the solid green line, which shows the corrected nomogram's actual situation. D: The models' clinical benefits were assessed using decision curve analysis. The black horizontal line indicates that when all patients do not receive treatment, regardless of the probability threshold, there is no clinical net benefit. The gray diagonal represents the change in clinical net benefit as the probability threshold changes when all patients receive treatment

The hematology model's AUC could reach 0.772 (95% CI: 0.699–0.845) Fig. 3 (Fig. 4A), which showed that hematological factors could effectively predict whether ESCC patients can achieve pCR after receiving nICT. The Youden index determined that -0.933 was the optimal threshold in the hematology model for differentiating among cohorts with non-pCR versus pCR (Fig. 3C). According to the actual classification, hemScores were significantly different among cohorts with non-pCR versus pCR (*P* < 0.001) (Fig. 3D).

### Construction and validation of the combined nomogram

We used univariate and multivariate analyses to analyze all clinicopathological factors to identify variables that were independently related to pCR (Table 3). Age, cN stage, tumor length, radScore, and hemScore were independent factors of pCR according to univariate analysis (*P* < 0.05); cN stage (cN0 was the reference quantity; cN1: *P* = 0.030; cN2: *P* = 0.012), radScore (*P* < 0.001), hemScore (*P* < 0.001), and tumor length (*P* = 0.028) had independent influences on pCR according to multivariate analysis. Therefore, we combined the above factors to construct a combined nomogram to predict whether ESCC patients could achieve pCR after nICT (Fig. 4B), which was also a demonstration of the results of multivariate analysis. Compared with those of the hematology model (AUC = 0.772, 95% CI: 0.699–0.845) and radiomics signature (AUC = 0.874, 95% CI: 0.819–0.928), the combined nomogram's AUC reached 0.934 (95% CI: 0.896–0.972), indicating a better ability for prediction (Fig. 4A). The DeLong test results demonstrated a difference in statistics in the diagnostic efficacy between the nomogram and the hematology model (*P* < 0.001) and the radiomics signature (*P* = 0.006), while there was also a statistical difference between radiomics and hematology models in diagnostic efficacy (*P* = 0.025). We evaluated the consistency of the combined nomogram model through a calibration curve (Fig. 4C) and the results of the Hosmer–Lemeshow test indicated a high degree of fit between the nomogram's actual and predicted date (*P* > 0.05). We also evaluated the clinical net benefit rates of the three models through DCA (Fig. 4D). The results showed that both the single and combined nomogram models could provide clinical benefits for patients within a certain risk threshold. In addition, when the combined nomogram model is used to guide clinical practice, it provides a greater clinical advantage to ESCC patients than the other models over a wide range of risks.

**Table 3:** Univariate and multivariate analyses of variables linked to pCR with ESCC patients

	**Univariate analysis**		**Multivariate analysis**	
**Variable**	***P***-value	**OR (95%CI)**	***P***-value	**OR (95%CI)**
Sex			-	-
Female	ref	ref		
Male	0.149	0.515(0.209,1.269)		
Age				
≤ 60	ref	ref	ref	ref
> 60	0.040*	2.100(1.035,4.262)	0.395	1.602(0.544,4.886)
cT stage			-	-
2	ref	ref		
3	0.393	0.592(0.177,1.973)		
4	0.486	0.560(0.105,2.986)		
cN stage				
0	ref	ref	ref	ref
1	0.019*	3.850(1.243,11.924)	0.030*	6.014(1.319,34.673)
2	0.048*	3.500(0.036,341.623)	0.012*	9.857(1.810,67.312)
cTNM stage			-	-
II	ref	ref		
III	0.092	2.111(0.886,5.033)		
IV	0.509	1.600(0.059,43.564)		
KPS score			-	-
80	ref	ref		
90	0.446	1.320(0.647,2.695)		
Tumor differentiation			-	-
High	ref	ref		
Low	0.358	0.571(0.173,1.886)		
Middle	0.297	0.542(0.089,3.279)		
Tumor location			-	-
Lower	ref	ref		
Middle	0.800	1.097(0.535,2.247)		
Upper	0.485	0.565(0.118–2.712)		
Smoking history			-	-
No	ref	ref		
Yes	0.881	0.950(0.487,1.853)		
Drinking history			-	-
No	ref	ref		
Yes	0.595	1.200(0.613,2.348)		
Tumor length	0.032*	0.757(0.587,0.976)	0.028*	0.619(0.395,0.935)
radScore	< 0.001*	17.674(6.678,46.779)	< 0.001*	23.555(7.983,92.744)
hemScore	< 0.001*	5.709(2.853,11.426)	< 0.001*	7.406(3.090,21.206)

Note. *cT* clinical T category, *cN* clinical N category, *cTNM* clinical TNM category, *KPS* Karnofsky performance status, * represented statistical significance

## Discussion

For locally advanced ESCC patients, when nCRT is combined with esophagectomy as the standard therapy, it is difficult to achieve a precise range during the treatment process, which inevitably causes radiation toxicity to surrounding tissues and organs, as well as an increased risk of complications such as esophageal fistula or death during the perioperative period. In this situation, nICT provides a novel possibility for the therapy of ESCC patients. According to the meta-analysis by Wang et al. [23], the security and effectiveness of nICT in treating esophageal cancer after surgery demonstrated that nICT in ESCC patients is safe and effective. The efficacy of nICT and nCRT can be maintained at the same level, while the safety of nICT is better than that of nCRT. The results of this research indicate that for ESCC patients, nICT may be the best choice for the current type of neoadjuvant therapy. In addition, a recent study showed that adding radiotherapy to immunochemotherapy can increase the incidence of grade 3–4 adverse effects compared to nICT (46.7% vs. 32.8%, *P* = 0.04), and the rate of pCR has not significantly improved, indicating that adding radiotherapy to nICT can significantly increase the risk of serious adverse events [24]. Therefore, nICT is still the most widely used treatment in clinical trials. However, there are significant differences in whether different patients can achieve pCR after nICT, thus making accurate stratification essential for the choice of therapy. In our study, we constructed a combined nomogram model that includes the radScore obtained from CT-based radiomics features, the hemScore from baseline hematological factors, and relevant clinicopathological factors. This model potentially identifies patients who will benefit from nICT and provides guidance or reference for individualized treatment.

In our study, the majority of patients used camrelizumab and sintilimab as immunotherapy drugs (97%). Although they are still in clinical trials for nICT in ESCC, they have been approved in China as immunotherapy drugs for advanced or metastatic ESCC. The chemotherapy combined with sintilimab or camrelizumab for ESCC has been approved as the therapeutic schedule of first-line, another thing to watch out for is that camrelizumab monotherapy has been approved as the therapeutic option for second-line [25–27]. According to current clinical trial results, camrelizumab and sintilimab as PD-1 inhibitors for nICT can achieve a pCR of 30%-50%, which is similar in efficacy to the PD-1 inhibitors recommended in the NCCN guidelines, indicating that the use of camrelizumab and sintilimab as PD-1 inhibitors for nICT, is supported by clinical trial evidence [10, 28, 29].

Radiomics as a hot technology has been shown to have satisfactory results in predicting patient treatment responses through numerous studies. Yang et al. [30] found that the AUC for predicting esophageal cancer pCR after nCRT based on CT radiomic signatures reached 0.79 (95% CI: 0.48–1.00) in the validation group. This indicates that radiomics provides more comprehensive information on intratumoral heterogeneity, can forecast the prognosis and effectiveness of patients and is expected to accurately guide the treatment of various solid cancers [31]. Liu et al. [32] utilized CT images for constructing and verifying a clinical-radiomics model for predicting a major pathologic response after nICT in non-small cell lung cancer (AUC = 0.81, 95% CI: 0.63–0.98). And beyond that, Liang et al. [33] generated an individual radiomics nomogram that included clinicopathological independent variables and radiomics features for predicting the response after PD-1 therapy in gastric cancer patients. The nomogram's AUC reached 0.778 (95% CI: 0.732–0.776), which was superior to that of the radiomics-only. Their findings demonstrate the value of radiomics for making treatment-related decisions for patients, especially with a more satisfactory predictive performance after incorporating the patient's clinicopathologic factors. Although radiomics is widely used in various medical fields, it is still in a state of continuous exploration. Due to the limited scope of segmentation, it cannot reflect the systemic states of patients. Therefore, our study included hematological factors, which are traditional and easily accessible, to more comprehensively reflect the actual condition of patients and to improve the predictability of patients who are sensitive to nICT. According to these results, the combined nomogram model with the inclusion of the hemScore had superior performance for predicting (AUC = 0.934, 95% CI: 0.896–0.972) in contrast with the radiomics signature, which proves that it is highly important to include hematology factors and that it provides plentiful patient information compared to primary clinicopathological factors.

In this study, we included 29 hematological factors and ultimately identified four factors to establish a hematological model, namely, LYM, ALB, HDL, and NLR, which have been reported in previous tumor immunotherapy studies. Research by Valero et al. [34] reported that the NLR with a high level is related to a reduced chance of immunotherapy response and a worse prognosis, suggesting a prognostic and predictive value of the NLR in immune checkpoint inhibitor (ICI) therapy. This may be due to the association of neutrophils with the tumor microenvironment. Some studies have reported that neutrophils are related to tumorigenesis, progression, and early dissemination [35, 36]. In contrast, lymphocytes, especially CD8 + T cells, play a vital function in antitumor response by causing the death of cytotoxic cells and preventing the migration and development of tumor cells [37, 38]. A decrease in the LYM may lead to inactivation of the tumor immune response, causing tumor progression and ultimately resulting in a poor prognosis [39]. A typical biomarker used to assess patients' nutritional status is the serum ALB level [40], which can indicate the inflammatory and immune conditions of patients. The results of Wu et al. [41] reported that patients with high serum ALB concentrations before immunotherapy had better survival. Perrone et al. [42] investigated the prognostic effects of factors linked to cholesterol on cancer patients, and their analysis showed that cholesterol passive diffusion (PD) levels were the only significant protective parameter for OS and PFS (*P* < 0.001, HR: 0.81). According to the authors, more mature HDL particles may be responsible for creating an inflammatory immunological environment that supports a better response to ICIs, which may explain the favorable correlations of cholesterol PD with OS and PFS. These studies show that the characteristics included in our hematology model are associated with the advancement and occurrence of cancers, as well as the response and prognosis of patients to treatment. Therefore, our hematology model based on these factors could be an accurate and a trustworthy reflection of the systemic condition of the patient. We also calculated the hemScore of the model and integrated it with the radScore, which reflects the patient's primary tumor status, to plot the combined nomogram associated with the patient's pCR, thus achieving a more accurate prediction of patient treatment response and providing trustworthy reference and guidance for individualized treatment of patients. Our study considered the clinical practicality evaluation of the model and plotted a DCA. The findings indicated that the combined nomogram model could provide potential clinical benefits for patients compared to any single model.

Several limitations of our study still exist. First, we did not explore the mechanism of pCR differences in ESCC patients after nICT. Second, because this study was retrospective, it is difficult to ensure the stability and consistency of the CT scanning parameters and image quality. Therefore, this study collected information only from non-contrast CT images to avoid differences in the development time and dose of contrast agents. Third, more than 30% of patients lacked some important tumor markers for ESCC, such as CA-199 and SCC. Therefore, these factors were not considered hematological factors. Finally, due to our small sample size, we did not further validate the model. In the future, our findings require independent verification with a larger number of samples to validate the applicability of the model as an effective auxiliary treatment decision-making tool.

## Conclusions

A combined nomogram model based on CT, hematological factors, and clinicopathological characteristics before treatment can effectively predict whether ESCC patients will achieve pCR after nICT, thus identifying patients who are sensitive to nICT and providing assistance for deciding on clinical therapy.

### Supplementary Information

**Supplementary Material 1. **

## Data Availability

The datasets generated or analyzed during the study are available from the corresponding author on reasonable request.

## Abbreviations

ESCC: Esophageal squamous cell carcinoma
pCR: Pathological complete response
nICT: Neoadjuvant immunochemotherapy
ICC: Intraclass correlation coefficient
DCA: Decision curve analysis
ROC: Receiver operating characteristic curve
nCRT: Neoadjuvant chemoradiotherapy
AUC: Area under the curve
LASSO: Least absolute shrinkage and selection operator
ALB: Albumin
HDL: High-density lipoprotein
LYM: Lymphocyte count
NLR: Neutrophil-to-lymphocyte ratio
TRG: Tumor regression grade
ROI: Region of interest
