# Supplementary material for: A combined nomogram based on radiomics and hematology to predict the pathological complete response of neoadjuvant immunochemotherapy in esophageal squamous cell carcinoma
Source: BMC Cancer. 2024 Apr 12;24:460. doi: 10.1186/s12885-024-12239-0 (PMC11015586; doi:10.1186/s12885-024-12239-0)
Supplement: Supplementary file 1 — Supplementary Material 1. [file 12885_2024_12239_MOESM1_ESM.docx]

**Supplementary material**

**Table S1**: Hematology factors and units of ESCC patients.

**Table S2**: LASSO regression selected hematology factor linked to pCR.

**Fig.S1:** Selection of radiomic features associated with pCR using the LASSO regression.

**Fig.S2:** Receiver operating characteristic curves of radiomics signature in actual data and balanced data with SMOTE.

**Fig.S3:** Selection of hematology factors associated with pCR using the LASSO regression.

**Table S1**: Hematology factors and units of ESCC patients.

| **Factor** | **Unit** | **Factor** | **Unit** |
| --- | --- | --- | --- |
| ALT | U/L | CLU | mmol/L |
| AST | U/L | CHOL | mmol/L |
| CK | U/L | HDL | mmol/L |
| LDH | U/L | LDL | mmol/L |
| HBDH | U/L | TBIL | umol/L |
| TP | g/L | SCr | umol/L |
| ALB | g/L | blood iron | umol/L |
| GLOB | g/L | LYM | X10^9^/L |
| HGB | g/L | NEU | X10^9^/L |
| FIB | g/L | MON | X10^9^/L |
| β2-MG | mg/L | PLT | X10^9^/L |
| CKMB | ng/ml | RBC | X10^12^/L |
| CEA | ng/ml | LMR | - |
| CYFRA21_1 | ng/ml | NLR | - |
| MCV | fL |  |  |

Note. ALT: alanine aminotransferase; AST: aspartate aminotransferase; CK: creatine kinase; LDH: lactate dehydrogenase; HBDH: α-hydroxybutyrate dehydrogenase; TP: total protein; ALB: albumin; GLOB: globulin; HGB: hemoglobin level; FIB: fibrinogen count; β2-MG: β2-microglobulin; CKMB: creatine kinase myocardial band; CEA: carcinoembryonic antigen; CYFRA21-1: cytokeratin 19 fragment antigen 21-1; MCV: mean corpuscular volume; GLU: glucose; CHOL: cholesterol; HDL: high-density lipoprotein; LDL: low-density lipoprotein; TBIL: total bilirubin; SCr: serum creatinine; LYM: lymphocyte count; NEU: neutrophil count; MON: monocyte count; PLT: platelet count; RBC: red blood cell count; LMR: lymphocyte-to-monocyte ratio; NLR: neutrophil-to-lymphocyte ratio.

**Table S2:** LASSO regression selected hematology factor linked to pCR.

| **Hematology factors** | **Coefcients** |
| --- | --- |
| LYM | -2.05839 |
| ALB | 0.015841 |
| HDL | -0.30136 |
| NLR | 0.344866 |

Note. LYM: lymphocyte count; ALB: albumin; HDL: high-density lipoprotein; NLR: neutrophil-to-lymphocyte ratio.


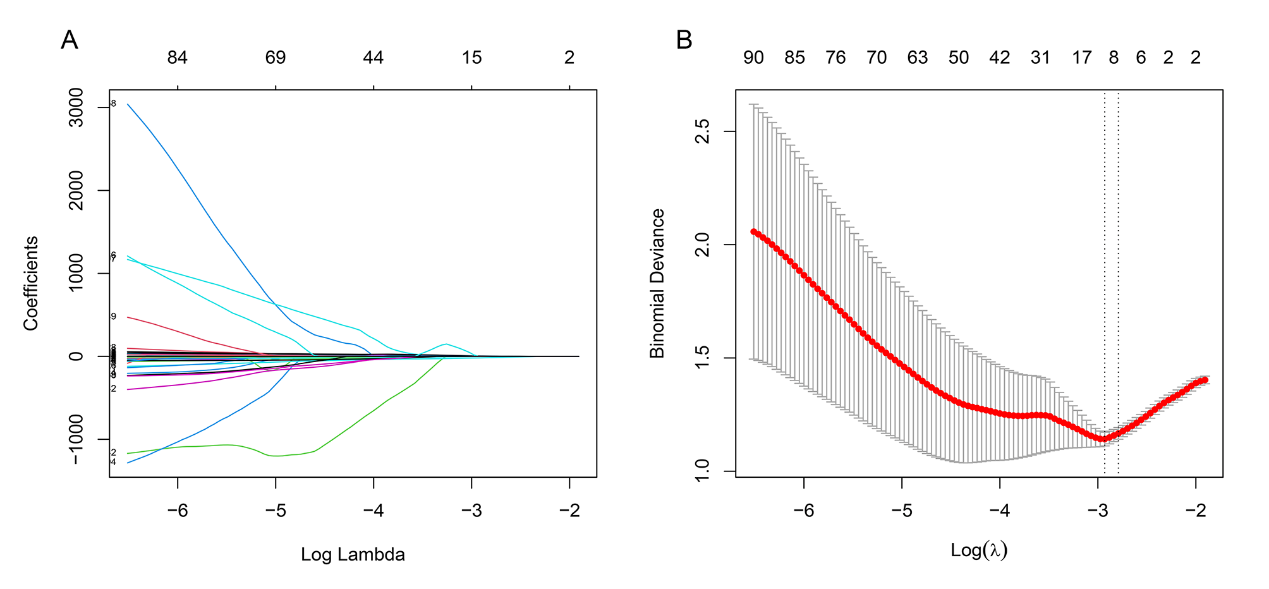


Fig.S1: Selection of radiomic features associated with pCR using the LASSO regression. A: Coefcients profles of radiomic features. The horizontal axis value is logλ, and the vertical axis value represent the coefcients of radiomic features. B: The cross-validation curve. The horizontal axis value is logλ, and the vertical axis value is partial likelihood deviance.


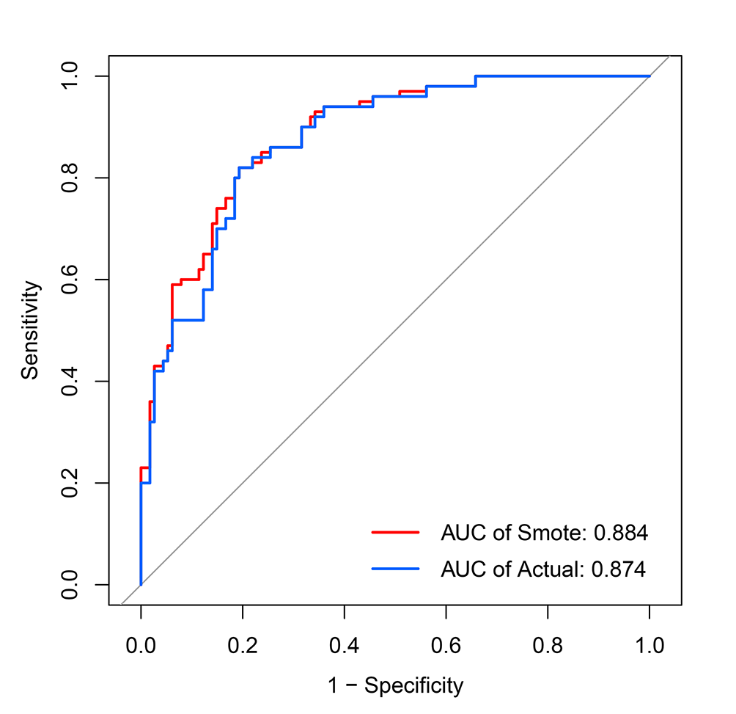


Fig.S2: Receiver operating characteristic curves of radiomics signature in actual data and balanced data with SMOTE. The AUC of actual data is 0.874 (95%CI: 0.819, 0.928), and the AUC of balanced data with SMOTE is 0.884 (95%CI: 0.841, 0.927).


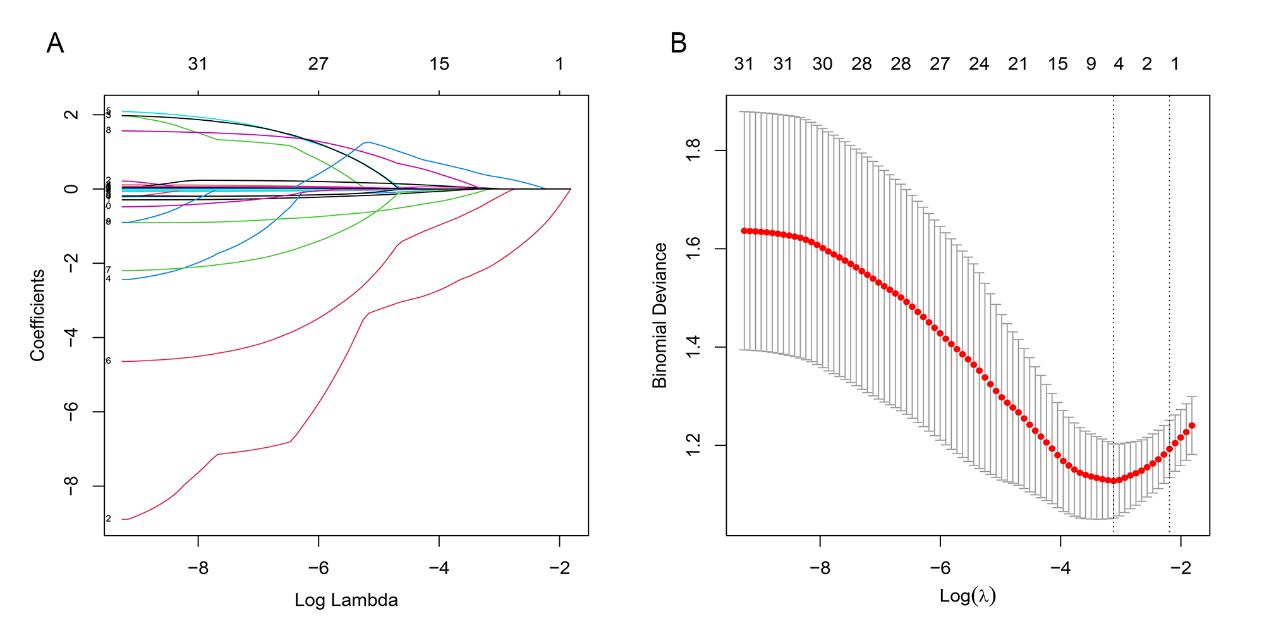


Fig.S3: Selection of hematology factors associated with pCR using the LASSO regression. A: Coefcients profles of hematology factors. The horizontal axis value is logλ, and the vertical axis value represent the coefcients of hematology factors. B: The cross-validation curve. The horizontal axis value is logλ, and the vertical axis value is partial likelihood deviance.
